# Supplementary material for: Succinivibrionaceae is dominant family in fecal microbiota of Behçet’s Syndrome patients with uveitis
Source: PLoS One. 2020 Oct 30;15(10):e0241691. doi: 10.1371/journal.pone.0241691 (PMC7598488; doi:10.1371/journal.pone.0241691)
Supplement: Attachment — Submitted filename: Response to Reviewers.docx [file pone.0241691.s001.docx]

**PONE-D-19-35822**

**Succinivibrio and Mitsuokella are Dominant Generas in Fecal Microbiota of Behçet’s Syndrome Patients with Uveitis**

**PLOS ONE**

Dear editor,

We revised the manuscript to address all the reviewer's comments in a point-by-point response. This rebuttal letter that responds to each point raised by the academic editor and reviewers.

Ayse Kalkanci

Corresponding author

**Editor’s comments:**

***1.    Please ensure that your manuscript meets PLOS ONE's style requirements, including those for file naming. The PLOS ONE style templates can be found at*** [***http://www.plosone.org/attachments/PLOSOne_formatting_sample_main_body.pdf***](http://www.plosone.org/attachments/PLOSOne_formatting_sample_main_body.pdf) ***and*** [***http://www.plosone.org/attachments/PLOSOne_formatting_sample_title_authors_affiliations.pdf***](http://www.plosone.org/attachments/PLOSOne_formatting_sample_title_authors_affiliations.pdf)

Style requirements were completed. DOI numbers were added if exists.

***2. In your Methods section, please state where the participants were recruited for your study.***

It was stated in methods section “Participants were recruited to our study at Gazi University Medical Faculty, Physical Medicine and Rehabilitation Department, Division of Rheumatology, Ankara, Turkey”.

***3. Please provide additional details regarding participant consent. In the ethics statement in the Methods and online submission information, please ensure that you have specified (1) whether consent was informed and (2) what type you obtained (for instance, written or verbal, and if verbal, how it was documented and witnessed). If your study included minors, state whether you obtained consent from parents or guardians. If the need for consent was waived by the ethics committee, please include this information.***

Informed written consent was obtained from each participant included in this study, according to the declaration of Helsinki.

***4. We note that you are reporting an analysis of a microarray, next-generation sequencing, or deep sequencing data set. PLOS requires that authors comply with field-specific standards for preparation, recording, and deposition of data in repositories appropriate to their field. Please upload these data to a stable, public repository (such as ArrayExpress, Gene Expression Omnibus (GEO), DNA Data Bank of Japan (DDBJ), NCBI GenBank, NCBI Sequence Read Archive, or EMBL Nucleotide Sequence Database (ENA)). In your revised cover letter, please provide the relevant accession numbers that may be used to access these data. For a full list of recommended repositories, see*** [***http://journals.plos.org/plosone/s/data-availability#loc-omics***](http://journals.plos.org/plosone/s/data-availability#loc-omics) ***or*** [***http://journals.plos.org/plosone/s/data-availability#loc-sequencing***](http://journals.plos.org/plosone/s/data-availability#loc-sequencing)***.***

Nucleotide suquences of 1797 taxa were deposited in NCBI GenBank with accession numbers MT573971 to MT575767 (file SUB7562864).

***5. Please include your tables as part of your main manuscript and remove the individual files. Please note that supplementary tables (should remain/ be uploaded) as separate "supporting information" files.***

Tables were included as a part of our manuscript.

***6. PLOS requires an ORCID iD for the corresponding author in Editorial Manager on papers submitted after December 6th, 2016. Please ensure that you have an ORCID iD and that it is validated in Editorial Manager. To do this, go to ‘Update my Information’ (in the upper left-hand corner of the main menu), and click on the Fetch/Validate link next to the ORCID field. This will take you to the ORCID site and allow you to create a new iD or authenticate a pre-existing iD in Editorial Manager. Please see the following video for instructions on linking an ORCID iD to your Editorial Manager account:*** [***https://www.youtube.com/watch?v=_xcclfuvtxQ***](https://www.youtube.com/watch?v=_xcclfuvtxQ)

ORCID iD for the corresponding author 0000-0003-0961-7325

***7. Please upload a copy of Figure 6, to which you refer in your text on page 7. If the figure is no longer to be included as part of the submission please remove all reference to it within the text.***

Reference of “Figure 6” was removed on page 7 and within the text.

***8. Please upload a new copy of Figure 2 as the detail is not clear. Please follow the link for more information:*** [***https://blogs.plos.org/plos/2019/06/looking-good-tips-for-creating-your-plos-figures-graphics/***](https://blogs.plos.org/plos/2019/06/looking-good-tips-for-creating-your-plos-figures-graphics/)

Figure 2 was revised and uploaded.

**Reviewer 1’s comments;**

***Reviewer #1: The authors conducted a metagenomic analysis in patients with BD and mined the species Prevotella copri from the data as a BD characteristic microbe. BD is rare and often difficult to treat even now and the finding may become very important data for the patients and treating physicians in the near future.

Major:
1. In the introduction section, the authors should describe detailed information of the differences in the “immunological pathologies” between BD patients with and without uveitis. We did not understand why the authors selected BD patients with uveitis for this study. If the authors need to use the data of FMF and CD as controls in this study, commonalities and differences in the “immunological pathologies” between BD and FMF, and between BD and CD would be helpful for us to understand more accurately. Discussion of the relationships between previous data of the immunological pathologies in patients with BD and metagenomic data of this study would be also important for the precise understanding.***

Introduction and discussion were both re-written including new comparative data BD&CD and BD&FMF. New references were added.

***2. The authors described their metagenomic data using OTU numbers in their figures and tables. Nonetheless, in their statistical analyses, the authors compared the metagenomic data using the taxonomic abundance. In the materials and methods section, the authors described that they used Kruskal-Wallis rank sum test to find significant differences in taxonomic abundance between patients and normal individuals (page 5). In the discussion section, the authors described that OTUs of this study groups were technically homogeneous (page 9). These descriptions were very confusing for us. If we understand it right, the authors used taxonomic abundance data for all statistical analyses of this study. We suggest that the authors utilize relative abundance titers of the metagenomic data for the comparison in the figures and tables with the statistical data. At least the figures and tables need the data of statistical analyses.***

We used relative abundance titers of the metagenomic data for the comparison in the figures and tables with the statistical data. Statistical analyses were added into the figures and tables. Materials and methods section was re-written for statistical analysis.

***3. The authors should provide us with BD patient demographical and clinical data with the statistical analyses. Gender (Front Immunol 2017; 8: 754.), aging (Cell Mol Life Sci 2018; 75: 129-148.), and medications (nonantibiotics, Nature 2018; 555: 623-8.) were reported to associate with gut microbe alteration. If the authors need to use the data of FMF and CD as controls in this study, the authors should provide us with the patients' data.***

Demographical and clinical characteristics of participants at the time of fecal sample collection were summaized at table 1

***4. High titers of alpha diversity in gut microbe composition were considered as a healthy state (Hepatology 2017; 65: 451-64.). The authors should describe the alpha diversity index titers of the 4 subgroups in this study with the detailed statistical analyses.***

Described.

***5. We need several references in the next two sentences for the precise understanding.
(1) Page 9: The molecular mimicry based on sequence homology between microbial and human peptides triggers autoimmune responses in BS patients.***

A new reference numbered by 16 was added “Hedayatfar A. Behçet's Disease: Autoimmune or Autoinflammatory?. J Ophthalmic Vis Res 2013; 8 (3): 291‐293”.

***(2) Page 10: We found Firmicutes and Proteobacteria were dominant phyla in BS patients presenting inflammatory effects in the gastrointestinal system.***

Two new references numbered by 18 and 19 were added “18. Shin NR, Whon TW, Bae JW. Proteobacteria: microbial signature of dysbiosis in gut microbiota. Trends Biotechnol 2015; 33 (9): 496-503” and “19. O Sheridan P, Martin JC, Lawley TD, et al. Polysaccharide utilization loci and nutritional specialization in a dominant group of butyrate-producing human colonic Firmicutes. Microb Genom 2016; 2 (2): e000043”.

***6. The authors should send the manuscript to the natives for revising the English.***

Revised.

***Minor:
1. We were wondering if the authors could provide us with the information of their minor revisions of the elusion steps in DNA extractions (Page 4).***
Minor revision at the elution step was performed by using 30 µl elution buffer.

***2. The authors should spell out the term of Treg in Page 9.***

“T regulator cell= Treg"

***Reviewer #2: The aim of this study was to investigate the gut microbiota of Behçet’s Syndrome patients with uveitis involvement and compare the gut microbiota of BS with that of Familial Mediterranean Fever and Crohn’s Disease. However, the following issues should be addressed.

1. The disease profiles of BS patients included in this study are unclear. Authors need to prepare the “Table” of patient’s data, such as the patients with BS (oral ulcer, skin lesion, genital ulcer, and so on).***

Demographical and clinical characteristics of participants at the time of fecal sample collection were summarized at table 1. Age were similar between groups (p: 0.098).

**Table 1. Demographical and clinical characteristics of participants at the time of fecal sample collection**

|  | Behçet’s Syndrome  (n:7) | Crohn’s disease  (n:9) | Familial Mediterranean Fever (n:12) | Healthy Controls  (n:16) |
| --- | --- | --- | --- | --- |
| Age, years | 35.57 $\pm$ 6.60 | 35.00 $\pm$ 5.27 | 32.17 $\pm$ 8.64 | 39.38 $\pm$ 7.69 |
| Men/women | 5/2 | 3/6 | 6/6 | 6/10 |
| **Disease characteristics** |  |  |  |  |
| Oral aphthosis, n (%) | 5 (71.43%) | 1 (11.11%) | 0 | 0 |
| Genital ulcers, n (%) | 1 (14.29%) | 0 | 0 | 0 |
| Skin lessions, n (%) | 3 (42.86%) | 1 (11.11%) | 0 | 0 |
| Uveitis, n (%) | 7 (100%) | 0 | 0 | 0 |
| Gastrointestinal system involvement, n (%) | 0 | 9 (100%) | 0 | 0 |
| Central nervous system involvement, n (%) | 1 (14.29%) | 0 | 0 | 0 |
| Vascular involvement, n (%) | 0 | 0 | 0 | 0 |
| Arthritis, % | 0 | 2 (22.22%) | 2 (16.67) | 0 |
| Pathergy positivity | 2 (28.57%) | NA | NA | NA |
| HLA-51 | 4 (57.14%) | NA | NA | NA |
| **Medications** |  |  |  |  |
| Colchicine, n (%) | 6 | 0 | 12 (100%) | 0 |
| Steroid, n (%) | 0 | 0 | 0 | 0 |
| 5-ASA/sulfasalazine, n (%) | 0 | 0 | 0 | 0 |
| Cyclosporine, n (%) | 1 | 0 | 0 | 0 |
| Azathioprine, n (%) | 4 | 9 | 0 | 0 |
| Methotrexate, n (%) | 0 | 0 | 0 | 0 |
| Infliximab, n (%) | 0 | 0 | 0 | 0 |

NA, not applicable

***2. Authors should also mention whether the patients were with active or inactive BS. When was the fecal sample obtained relative to the onset of an attack?***

The fecal samples of patients with BS were obtained at the active uveitis attack.

***3. Did the patients included in this study receive medication before the fecal sample obtained? Immunomodulatory drugs and steroids would impact on the gut microbiota composition.***

Informations about medication at the time of sample collection were summarized at table 1.

***4. The detailed information of control was also unclear. The human gut microbiota composition will differ from the age, sex, life style, diet and inflammatory status of donors (see PMID: 24705962 and 20498852). Thus, author should provide more profiles of patients and healthy controls, such as their age and sex. And author should mention whether the healthy control were with diabetes, cardiovascular diseases, systemic disease and other inflammatory diseases.***

16 healthy controls; 6 male (39-50y) and 10 female (24-52y) matched for age and sex. Patients were excluded from the study if they had any of the following: patient suspected to have BD clinically but did not fulfil the inclusion criteria; pregnancy, chronic diseases, psychiatric disorders, cancer, diabetes, cardiovascular diseases, systemic disease and other inflammatory diseases, dependence on alcohol or other substances. Dietary behaviors were similar in BS, FMF, CD and HC groups.

Demographical characteristics including age and sex of all participants were summarized at table 1. Individuals without systemic disease and inflammatory disease were included as healthy controls.

***5. The data regarding the abundant taxa at the family level in four groups suggested Succinivibrio and Mitsuokella were dominant and signature families, whereas Bacteroides was absent in BS patients. However, the statistical analysis were without correction. The statistical analysis in the genera and species levels also required correction.***

All data was corrected and marked.

***6. The authors are aware of the limitations of this study. However, mono-colonization of dominant species or fecal transplantation from BS patients in germ-free mice would also be informative.***

Information about the dominant fungal species obtained from BS patients was added into discussion. (Ye Z, Zhang N, Wu C, Zhang X, Wang Q, Huang X, Du L, Cao Q, Tang J, Zhou C, Hou S, He Y, Xu Q, Xiong X, Kijlstra A, Qin N, Yang P. A metagenomic study of the gut microbiome in Behcet's disease. Microbiome 2018; 6 (1): 135.

***7. The OTU clustering methods need to be clarified. The version of the software and algorithms used also need to be indicated. It is also unclear how the authors performed taxonomic assignment or what database they used.***

Obtained sequences were transferred into Operational Taxonomic Units (OTUs). Similarity of 99% was accepted as same phylotype. Each value was expressed as mean ± SD and a p value less than 0.05 was considered significant.

Statistical analysis was performed using R (Ver 3.6.0,, GPL-3 license). The relative abundances (expressed as parts per unit, calculated with ade4 packet in R, were directly compared in each taxonomic (for different phylum, genus, species) levels. vegan package was used for alpha biodiversity. Alpha diversity is defined as the diversity within a community and is mainly measured by Chao 1 diversity indexes. Chao 1 index estimates richness in numbers of taxa frequencies. The microbial diversities data were tested by using Wilcoxon rank sum test in Past 3.20 (Øyvind Hammer, 2018). Based on relative abundance features, R packages FactoMineR and factoextra were used for dendrogram (Bray-Curtis similarity) and taxa distribution visualizations with groups while ggfortify and ggplot2 were used for PCA.

***8. Minor issue, see Page 3, lines 17, "Behçet's syndrome" should be used as abbreviation "BS"***

Corrected

***9. Minor issue, see Page 11, lines 1, "Prevotella" should be used in italic.***

 Corrected
