# Supplementary material for: Succinivibrionaceae is dominant family in fecal microbiota of Behçet’s Syndrome patients with uveitis
Source: PLoS One. 2020 Oct 30;15(10):e0241691. doi: 10.1371/journal.pone.0241691 (PMC7598488; doi:10.1371/journal.pone.0241691)
Supplement: Attachment — Submitted filename: Response to Reviewers for R4.docx [file pone.0241691.s002.docx]

**PONE-D-19-35822**

# *Succinivibrionaceae* is Dominant Family in Fecal Microbiota of Behçet’s Syndrome Patients with Uveitis

**PLOS ONE**

Dear editor,

We revised the manuscript to address all the reviewer's comments in a point-by-point response. This rebuttal letter that responds to each point raised by the reviewers.

Ayse Kalkanci

Corresponding author

***1) Please ensure that you refer to Figure 6 in your text as, if accepted, production will need this reference to link the reader to the figure.***

Figure 6 was referred in the text.
