# Supplementary material for: Succinivibrionaceae is dominant family in fecal microbiota of Behçet’s Syndrome patients with uveitis
Source: PLoS One. 2020 Oct 30;15(10):e0241691. doi: 10.1371/journal.pone.0241691 (PMC7598488; doi:10.1371/journal.pone.0241691)
Supplement: Attachment — Submitted filename: Response to Reviewers for R7.docx [file pone.0241691.s003.docx]

**Response to the reviewers**

***"Succinivibrionaceae is Dominant Family in Fecal Microbiota of Behçet’s Syndrome Patients with Uveitis"***

*1) Please amend your Response to Reviewers letter to include a point by point response to each of the points made by the Editor and / or Reviewers. Please follow this link for more information: http://blogs.PLOS.org/everyone/2011/05/10/how-to-submit-your-revised-manuscript/*

Answer: Yes . “Response to reviewers-7” letter was added into submission.

*2) Thank you for providing your revised Funding statement. We've made some updates to conform to journal requirements. Can you please confirm in your Cover Letter whether the following proposed statement is accurate and suitable to appear alongside your manuscript?*

*"This project was supported and funded with a grant of Turkish Society of Physical Medicine and Rehabilitation. The funder had no role in study design, data collection and analysis, decision to publish, or preparation of the manuscript."*

*If there are any errors or omissions in this statement please let us know. Otherwise, please include the above statement in your Cover Letter upon re-submission, and we will update the statement in the submission form on your behalf.*

Answer: "This project was supported and funded with a grant of Turkish League Against Rheumatism. The funder had no role in study design, data collection and analysis, decision to publish, or preparation of the manuscript." This statement included in my Cover Letter.
